# On-line anxiety level detection from biosignals: Machine learning based on a randomized controlled trial with spider-fearful individuals

**DOI:** 10.1371/journal.pone.0231517

**Published:** 2020-06-23

**Authors:** Frank R. Ihmig, Antonio Gogeascoechea H., Frank Neurohr-Parakenings, Sarah K. Schäfer, Johanna Lass-Hennemann, Tanja Michael

**Affiliations:** 1 Department of Biomedical Microsystems, Fraunhofer-Institut für Biomedizinische Technik IBMT, Sulzbach/Saar, Germany; 2 Faculty of Electrical Engineering, Mathematics and Computer Science, University of Twente, Enschede, The Netherlands; 3 Division of Clinical Psychology and Psychotherapy, Department of Psychology, Saarland University, Saarbrücken, Germany; Politechnika Krakowska im Tadeusza Kosciuszki, POLAND

## Abstract

We present performance results concerning the validation for anxiety level detection based on trained mathematical models using supervised machine learning techniques. The model training is based on biosignals acquired in a randomized controlled trial. Wearable sensors were used to collect electrocardiogram, electrodermal activity, and respiration from spider-fearful individuals. We designed and applied ten approaches for data labeling considering individual biosignals as well as subjective ratings. Performance results revealed a selection of trained models adapted for two-level (low and high) and three-level (low, medium and high) classification of anxiety using a minimal set of six features. We obtained a remarkable accuracy of 89.8% for the two-level classification and of 74.4% for the three-level classification using a short time window length of ten seconds when applying the approach that uses subjective ratings for data labeling. *Bagged Trees* proved to be the most suitable classifier type among the classification models studied. The trained models will have a practical impact on the feasibility study of an augmented reality exposure therapy based on a therapeutic game for the treatment of arachnophobia.

## Introduction

About 7.4% of the population meets the criteria of a specific phobia at least once in their lifetime [1]. The pathological fear of spiders is one of the most common specific phobias. When patients are confronted with the phobic object, they react with strong physical anxiety symptoms such as tachycardia, sweating or shortness of breath.

Exposure therapy is the method of choice for the treatment of specific phobias [2]. During exposure, the patient is confronted with the feared object under controlled conditions. Exposure therapy may be conducted *in vivo*, i.e. the patient is confronted with the phobic stimuli in reality, or *in sensu*, i.e. the patient is confronted with the phobic object in his imagination. Exposure therapy is highly successful [3], nevertheless, it is rarely used in routine clinical care. One barrier is its practicability as it involves high organizational and logistical efforts.

Virtual reality exposure therapy (VRET), which started about 20 years ago [4], and augmented reality exposure therapy (ARET) are promising alternatives to *in vivo* or *in sensu* exposure [5–7]. They have already been shown to be effective in several anxiety disorders, such as fear of flying [8], social phobia [9] and spider phobia [10] as well as posttraumatic stress disorder [11,12]. Besides the smaller logistical effort, *in virtuo* therapy offers increased control during sessions, allowing therapists to manipulate the characteristics, frequency and intensity of exposure [13,14].

Moreover, the integration of gamified elements into VRET/ARET can increase patients’ engagement [15] and may reduce the experienced subjective distress [16]. Such serious games are gaming technologies aiming to entertain but also to educate, inform and train [17,18]. In a VRET/ARET system for spider phobia, biofeedback on the level of anxiety could be used to modulate serious game elements. Moreover, such a closed-loop system would allow a better monitoring (progress and safety) and individualized treatments.

The focus of this research is the usage of physiological responses for anxiety level detection. The Autonomic Nervous System (ANS) produces physiological responses to regulate body functions, such as heart activity. Important physiological responses related to stress and anxiety can be derived from electrocardiogram (ECG), electrodermal activity (EDA), and respiration (RSP) signals [18].

Heart rate (HR) and heart rate variability (HRV) can be extracted from ECG signals. While HR represents the number of heart beats per minute, HRV reflects the variation in time between consecutive heart beats. Lower HR is associated with relaxation and resting periods, whereas higher HR is related to disturbance and emotional arousal [19]. Contrary to HR, HRV increases during resting periods and decreases during stress. Apart from this, ECG feature extraction is used in current research to detect abnormal heart conditions [20] and for the development of human authentication systems [21].

Respiration can also be affected by emotional stimuli and is well-known as an indicator of psychological stress and anxiety [22,23]. Similar to HR, breathing rate (BR) increases as the levels of stress or anxiety increase leading to hyperventilation in extreme cases [24]. BR decreases with relaxation, while tense situations may cause interruptions in respiration. It is calculated by counting the number of breathing cycles per minute. The monitoring of breathing patterns has also been proposed as a way to affect the oscillations in HRV due to respiration [25]. This is because of the close relationship between respiratory and cardiovascular processes known as the respiratory sinus arrhythmia.

EDA is a measure of changes in the electrical conductance of skin based on the production of sweat. It is widely used as an indicator of psychological stress and anxiety [22,26]. This physiological response consists of two components: skin conductance level and skin conductance response. Skin conductance level is the tonic level that slowly varies over time. Skin conductance response is the phasic response to an emotionally arousing stimulus that is reflected in faster variations of the skin conductance level.

Interestingly, results from traditional psychophysiology studies (i.e. without using machine-learning techniques) often show no significant correlations between physiological measures and subjectively rated anxiety levels. Correspondingly, a recent meta-analysis reported that “there is no one-to-one mapping between an emotion category and a specific ANS response pattern” [27].

However, other research using machine-learning techniques show that emotion recognition through the analysis of physiological responses is feasible. We report similar studies to our work in the next section. So-called *supervised learning* consists of training an algorithm to approximate a model that predicts new unseen input features. *Supervised* refers to the fact that a human expert provides feedback for the algorithm training by labeling input data and thus, creating targets. Since the labeling is based on assumptions, a certain degree of bias is inevitable.

**Fig 1:**
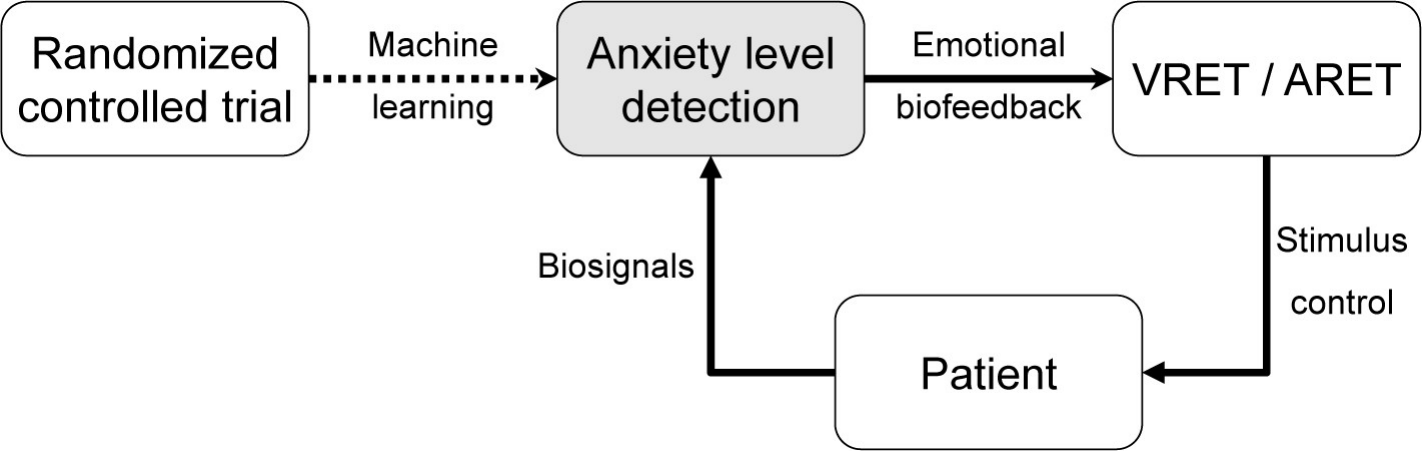
Closed-loop system architecture. Use of anxiety level detection to control stimulus intensity in the VRET/ARET setting enabling individualized treatment.

This paper describes the algorithm development for on-line anxiety level detection from biosignals recorded in a randomized controlled trial (RCT) for the envisioned use as emotional biofeedback in therapeutic games. Fig 1 shows a closed-loop system architecture using anxiety level detection to control stimulus intensity in a VRET/ARET setting enabling individualized treatment.

The objective is to compare different classification models to identify the most suitable classifier type that fulfils the VRET/ARET requirements. Since the development of the algorithm is of practical relevance, the following requirements are essential for future implementation of a portable and affordable VRET/ARET system with on-line anxiety level detection capability:

- Use of low-cost commercial wearable sensors to acquire relevant biosignals.
- Use of a minimal set of features to reduce computing complexity and processing power.
- On-line prediction of two or three levels of anxiety with an update rate of 10 seconds.
- Integration into a Microsoft Windows desktop application using.net assembly.
- Capability for future self-help and minimal-contact therapies.

Based on our literature search, we found a lack of an appropriate dataset that is close to our envisioned application (VRET/ARET system). There are several datasets publicly available that are related to stress detection but not explicitly to anxiety detection. Also, it is of advantage to have a similar stimulus and the same sensor hardware for dataset compilation as in the envisioned application. These were the reasons why we decided to conduct an RCT with spider-fearful individuals using a similar stimulus and the same sensor hardware to record and compile a dedicated dataset for our research and development purpose.

Furthermore, the scientific aim is to deliver new findings for automated anxiety level detection based on biosignals using state-of-the-art methods of pattern recognition and statistical learning. The following research questions will be answered to fulfil this aim:

- Which are the most relevant biosignal features?
- Which is the most suitable classifier?
- How is the accuracy of classifiers influenced by the combination of biosignal features?
- How is the accuracy of classifiers affected by the use of different time window lengths?

The structure of this paper is organized as follows: the second section introduces related work. The third section describes the recorded dataset, feature extraction and classifier training. The fourth section describes the evaluation results of the trained machine learning algorithms. Section five discusses our results in comparison with related work and gives recommendations for future research. Finally, the sixth section concludes our paper with a summary of the main contributions.

## Related work

To date, several supervised machine-learning techniques [28–32] have proven to be appropriate for stress and anxiety detection, e.g. support vector machines (SVM), neural networks, naïve Bayes, discriminant analysis, and decision trees.

Healey and Picard [28] reported the highest accuracy of 97.4% using a linear discriminant classifier when collecting several physiological markers [electromyogram (EMG), ECG, EDA, RSP and video] from 24 drivers in the Greater Boston area. Data were categorized into three different levels of stress: low (resting phase at the beginning and the end), moderate (highway), and high (cities). Keshan et al. [29] and Chen et al. [30] used the same paradigm applying different classifiers (various classifiers vs. one classifier) and window lengths (5 minutes vs. 10 seconds). The findings of Chen et al. reveal that features obtained from ECG, EDA and RSP can be satisfactory to achieve an accuracy rate of 89% for stress detection using an SVM classifier. Whereas Keshan et al. used various classifiers (naïve Bayes, neural networks, and decision trees) to obtain an accuracy of about 70% to categorize three levels of stress.

Additionally, Barua et al. [31] retrieve different physiological measures obtained from respiration and finger temperature sensors using a time window of 60 seconds. The study explores three different classifiers (neural networks, SVM, and case-based reasoning) to differentiate two levels of stress, with case-based reasoning achieving the highest accuracy of 85.6%. In a related VRET experiment, Handouzi et al. [32] intended to differentiate two levels of anxiety in seven participants with social phobia. Anxiety was induced using six different VR scenarios and features were extracted from blood volume pulse signal. An SVM algorithm with a time window of 20 seconds reached a 76% accuracy for the differentiation of two anxiety levels.

## Materials and methods

### Study protocol and dataset

**Fig 2:**
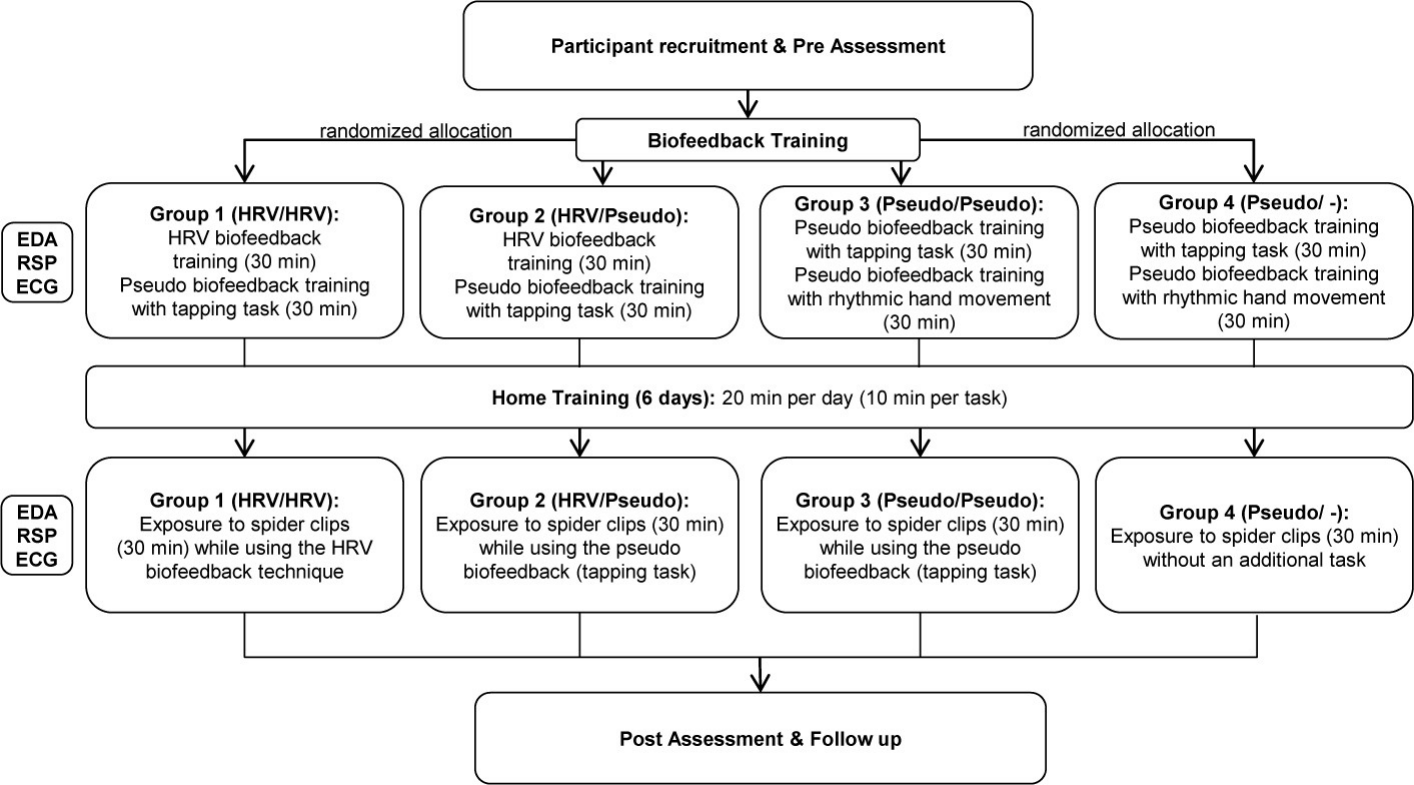
Simplified study flow chart of the randomized controlled trial adapted from Schäfer et al. [33].

The classifier development is based on supervised machine learning techniques using a dataset recorded in an RCT with 80 spider-fearful individuals aged between 18 and 40 years. The trial itself (German Clinical Trials Register DRKS00012278, registered on 23 May 2017, amendment on 5 October 2017) is not part of this work, details of the study protocol are described in Schäfer et al. [33]. Ethical approval for the trial has been granted by the Ethical Committee of the Faculty of Human Science of Saarland University (reference: 17–03). Participants provided their written informed consent. Consent for publication has been obtained from all participants as part of the informed consent process. The main focus of this RCT was to investigate if the use of an HRV biofeedback intervention could be a promising therapeutic add-on to exposure therapy for specific phobias [34,35]. Furthermore, biosignal measurements (EDA, ECG and RSP) were recorded during the biofeedback training session and the exposure session and were analyzed according to the data labeling approaches described later. Fig 2 shows the simplified study flow chart.

**Fig 3:**
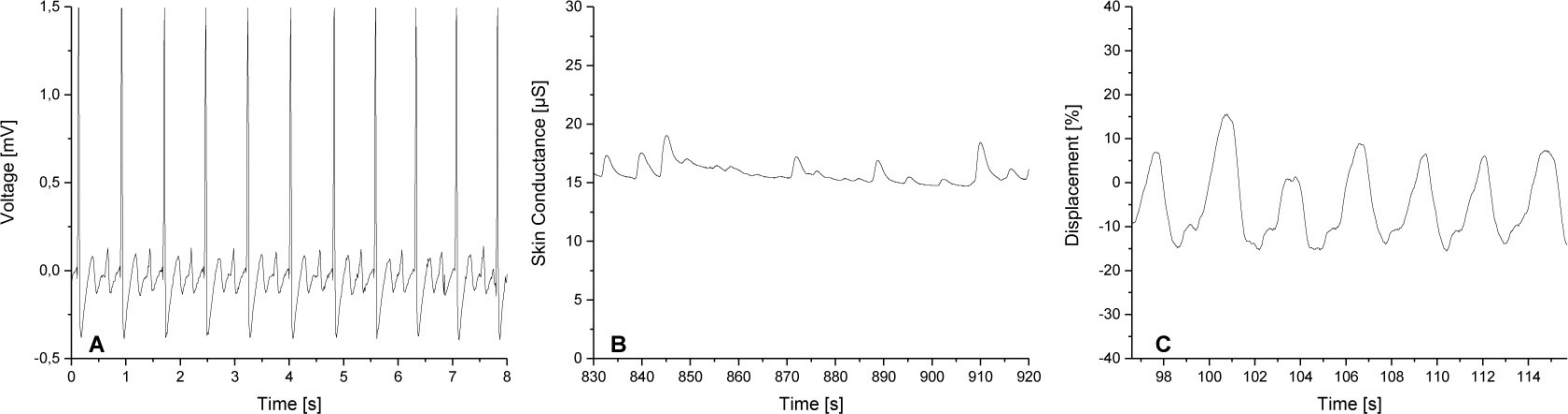
Schematic illustration of the exposure procedure including time points of arousal assessment.

All subjects were introduced to the exposure procedure and rationale that is mainly based on the principles of the one-session exposure treatment developed by Öst [36]. All experimental groups received a biofeedback training session starting with a 5-minute resting phase. During the biofeedback training session, they learned two tasks, either HRV biofeedback and a pseudo-biofeedback task or two pseudo-biofeedback tasks. After one week of home training, all participants returned and watched a series of spider video clips. Each session started with a 1-minute demo clip followed by 16 1-minute spider video clips, all taken from TV documentaries showing detailed shots of spiders, and ended with a 5-minute resting phase. The sixteen clips with spiders were divided into two groups: clips 1–8 and clips 9–16. The order of the clips within each group was randomized. After every fourth video clip, participants were asked to rate their subjective arousal levels on 4-point scales ranging from “1 = not at all” to “4 = strongly”. Fig 3 shows a schematic illustration of the exposure procedure.

Subjects were divided into four groups (see Fig 2), of which only group 1 utilizes the HRV biofeedback during exposure. Consequently, the dataset of the exposure session of groups 2, 3, and 4 was used for classifier training. The records of group 1 were not considered, since the subjects applied a trained breathing technique. Thus, our dataset for classifier training and validation contains records from 57 out of 60 subjects. Each record is approximately 35 minutes long. Three records were disregarded due to technical problems during data acquisition.

**Fig 4:**
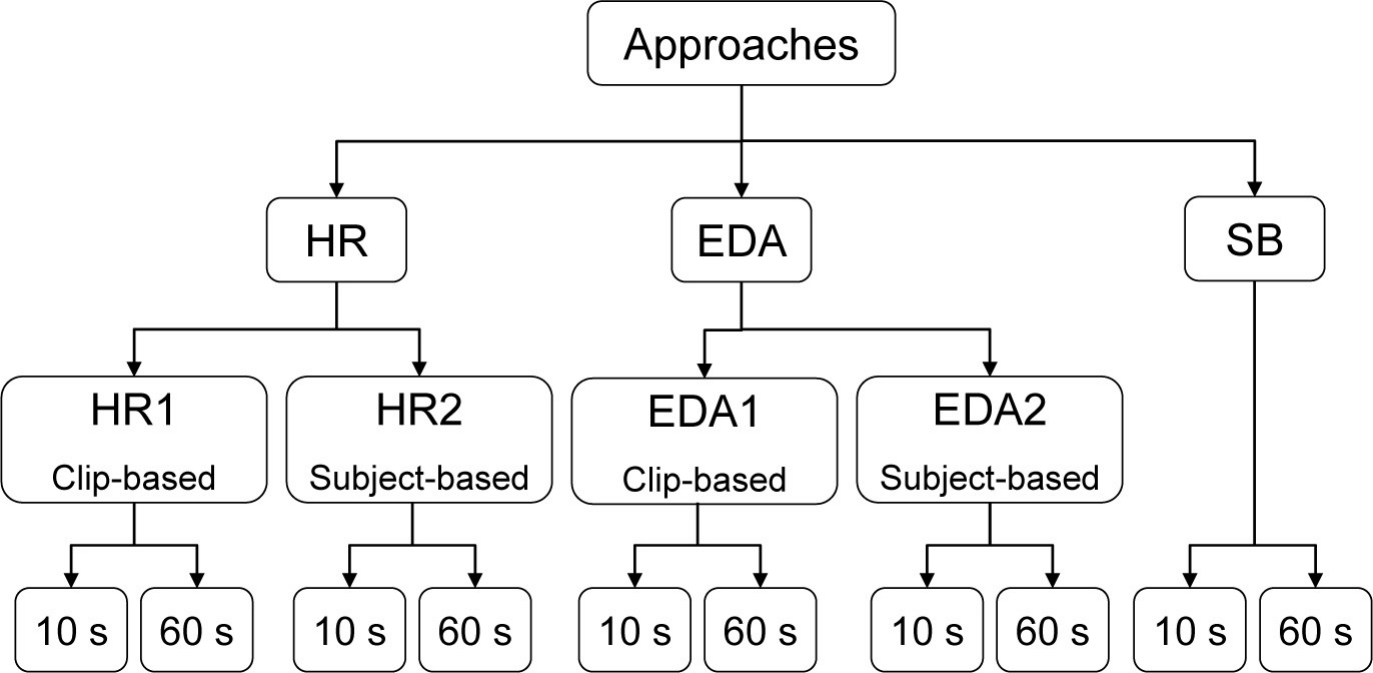
Overview of data labeling approaches.

We designed ten different approaches to extract biosignal features. These were defined by the way of labeling the clips during each data record to differentiate between two (low and high) or three levels/classes (low, medium and high) of anxiety. As can be seen in Fig 4, the main ones are HR, EDA, and subjective (SB) approaches. The SB approach is based on self-rated arousal during the exposure. Both, HR and EDA approaches are subdivided into clip-based (HR1 and EDA1) and subject-based (HR2 and EDA2) approaches. The last ramification of each sub-approach corresponds to the length of the time window for feature extraction: 10 seconds as the envisioned update rate for on-line prediction and 60 seconds for performance comparison of the classifiers.

#### HR and EDA approaches

It is expected that HR and EDA will increase during the most disturbing clips. Given this assumption, clip-based and subject-based approaches were defined. The clip-based approaches (HR1 and EDA1) establish labels to the video clips by sorting the average of the normalized signal of all the records. The three clips with the highest normalized mean for HR and EDA were labeled as 'high' and three other clips with medium average values were labeled as 'medium'. As for the 'low' label, the last 3 minutes of the resting phase from the biofeedback training session were picked as it is assumed that during this phase the subjects were the most relaxed. The main advantage of this approach is that the data for classifier training is balanced (i.e. same number of observations per class). However, at the same time, it assumes that the same clip is equally disturbing for all subjects, which is not plausible, and may bias the approach.

On the other hand, the subject-based approaches (HR2 and EDA2) establish labels to the video clips by considering individual responses. From the 16 clips, the 8 clips with the highest individual normalized mean for HR and EDA were labeled as 'high' and the remaining 8 clips were labeled as 'medium'. As for the 'low' classification, the entire 5-minute resting phase from the biofeedback training session was chosen to achieve the most balanced observation. However, the disadvantage of this approach is that data is still not totally evenly distributed (High-38%, Medium-38%, Low-24%).

#### SB approach

The SB approach is based on subjective arousal ratings during exposure. Labeling was done as follows: 'high' corresponds to the two highest ratings 3 and 4, whereas 'medium' corresponds to the two lowest ratings 1 and 2. With regard to the 'low' category, the last 3 minutes of the resting phase from the biofeedback training session were selected (equal to the clip-based approaches). As arousal ratings were obtained at four time points, only data of the clip right before each rating is taken into account. The disadvantage of this approach is unbalanced data as most of the subjects unevenly rated the clips and some rated their arousal stable across all points of assessment. Moreover, the approach only considers four clips for either 'high' or 'medium' and 3 minutes for 'low'. Table 1 displays a summary of the number of observations per approach.

**Table 1:** Summary of the number of observations per approach.

Approach	Number of observations			
	60 s		10 s	
	2 levels	3 levels	2 levels	3 levels
SB	283	398	1702	2392
HR1/EDA1	341	512	2051	3075
HR2/EDA2	740	1196	4439	7170

SB, subjective approach; HR1/EDA1, clip-based approaches; HR2/EDA2, subject-based approaches.

### Biosignal processing and feature extraction

**Fig 5:**
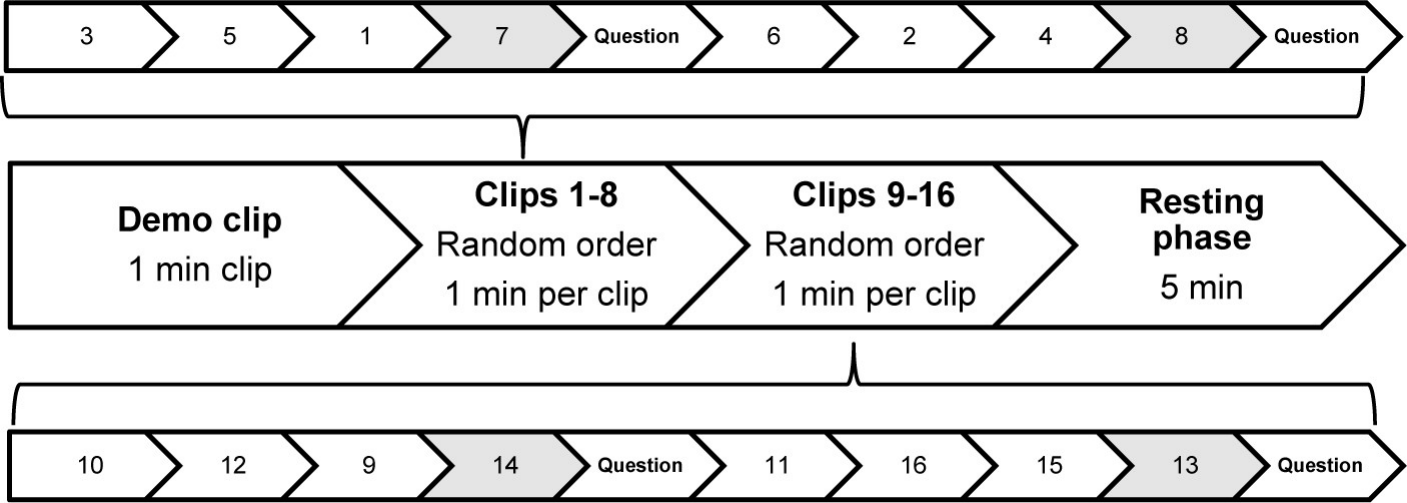
Exemplary plots of the three biosignals. Raw data acquired using the *BITalino* device (100 Hz sample rate): (A) Voltage for ECG processing, (B) Skin conductance for EDA processing, and (C) piezo sensor displacement for RSP processing.

Biosignals were recorded using IBMT’s Biofeedback System (BFS), which is described in Schäfer et al. [33]. This system supports state-of-the-art wearable sensors and wireless communication. In this RCT, the *BITalino* biosignal measurement device (PLUX–Wireless Biosignals S.A., Lisbon, Portugal) was used to record ECG, EDA and RSP signals with the sampling frequency set to 100 Hz per channel with 10-bit resolution, which is sufficient for ECG rhythm monitoring [37]. Three electrodes are placed according to standard lead II configuration for ECG measurement. Two electrodes are attached to the proximal part of the palm of the participant’s nondominant hand for EDA measurement. The electrodes used are standard pregelled and self-adhesive disposable Ag/AgCl electrodes (Kendall H135SG, Medtronic, Minneapolis, MN, USA). The RSP sensor is an adjustable, elastic-fastening chest strap with an integrated piezoelectric sensor. Fig 5 shows exemplary plots (raw data) of the three biosignals acquired using the *BITalino* device.

Both, the biosignal processing and feature extraction were developed in MATLAB R2017b (The MathWorks Inc., Natick, MA, USA). In total, 25 statistical and signal-specific features in time domain were extracted from each biosignal (Table 2). Frequency domain analysis was disregarded as the window lengths of the present study are too short to allow for accurate spectral analysis. According to the European Heart Journal, accurate short-term power spectral analysis requires window lengths of two to five minutes [38].

**Table 2:** Summary of 25 statistical and signal-specific features in time domain.

Electro-cardiogram	Respiration	Electrodermal Activity
Nmean	Nmean	Nmean
std	std	std
NFD	NFD	NFD
NSD	NSD	NSD
HRV	BRV	nOR
avNN	avNN	mmOR
sdNN	sdNN	mdOR
rMSSD		
NN50		
pNN50		
pNN20		

Statistical features are shaded light gray. Feature abbreviations are explained in Eqs 1–15.

Concerning the ECG signal, the MATLAB function of the Pan-Tompkins QRS detection algorithm [39], implemented by Sedghamiz [40], was used to extract the HR and Normal-to-Normal Interval (NNI) values. In general, this algorithm follows six steps:

1. Bandpass filtering (5–12 Hz) to eliminate noise and artifacts.
2. Differentiation to obtain the slope of the QRS complexes.
3. Squaring the last step to highlight the slope.
4. Moving-window integration to obtain waveform feature information in addition to the slope.
5. Adaptive thresholding since the peaks are variable.
6. Decision rule algorithm to distinguish between true and false peak detection.

A baseline for each subject is necessary to normalize the mean HR. It was calculated by taking the mean of the HR during the resting phase of the biofeedback training session (see Eq 1).

Regarding the RSP signal, it is acquired as the sensor displacement value in percentage, thus, the calculation of the BR was done by counting the number of times that the chest rises (equivalent to number of peaks). A band-pass filter with cutoff frequencies (0.1–24 Hz equivalent to 6–24 breaths per minute) was implemented to eliminate the offset and high-frequency noise. Moreover, the peaks were found using the MATLAB function *ndpeaks*. The baseline for the normalization of the mean BR was obtained in the same way as for HR (see Eq 1).

Concerning the EDA signal, a second order Butterworth low-pass filter with a cutoff frequency of 1.5 Hz was computed for the extraction of the statistical features. As for the signal-specific skin conductance orienting responses, further steps were implemented to obtain them according to the recommendations of Braithwaite et al. [41]: First, a high-pass filter with a cutoff frequency of 0.05 Hz was applied to produce a phasic signal. Then, the onset, offset and peaks are detected with a threshold of 0.03 Siemens. The number of orienting responses is simply the number of peaks detected (Eq 13), the mean magnitude of orienting responses is the difference between the magnitude of the peak and its respective onset (Eq 14), and the mean duration of orienting responses is the difference in time between the onset and the offset (Eq 15). The baseline for the normalization of the mean EDA is also obtained from the resting phase of the biofeedback training session but, in this case, Eq 2 proposed by Lykken et al. [42] was used instead of averaging the EDA signal.

Four statistical features were extracted from each biosignal that correspond to the ones proposed for emotion recognition by Picard et al. [43]:

- Normalized mean (*Nmean*):
  $$HR/BR:\;Nmean=\frac{1}{N}\sum_{n=1}^{N}\left(x_{n}-\mu_{t}\right)$$ (1)
  $$EDA:\;Nmean=\frac{1}{N}\sum_{n=1}^{N}\frac{x_{n}-\min(x_{t})}{\max(x_{t})-\min(x_{t})}$$ (2)
- Standard deviation (*std*):
  $$std=\sqrt{\frac{1}{N-1}\sum_{n=1}^{N}{(x_{n}-\mu_{x})}^{2}}$$ (3)
- Mean of the absolute values of the Normalized First Differences (*NFD*):
  $$NFD=\frac{1}{N-1}\sum_{n=1}^{N-1}\left|{\hat{x}}_{n+1}-{\hat{x}}_{n}\right|$$ (4)
- Mean of the absolute values of the Normalized Second Differences (*NSD*):
  $$NSD=\frac{1}{N-2}\sum_{n=1}^{N-2}\left|{\hat{x}}_{n+2}-{\hat{x}}_{n}\right|$$ (5)
  where *x_n_* represents the n^th^ sample of the corresponding signal, ${\hat{x}}_{n}$ the n^th^ normalized sample of the corresponding signal, *x_t_* the signal during the resting phase of the biofeedback training session, *N* the total number of samples, μ_*x*_ the mean of the signal during the current window, and μ_*t*_ the mean of the signal during the resting phase of the biofeedback training session.

Regarding HRV, the following seven features were investigated:

- Heart Rate/Breathing Rate Variability (*HRV/BRV*):
  $$HRV/BRV=\frac{1}{N}\sum_{n=1}^{N-1}({NNI}_{n+1}-{NNI}_{n})$$ (6)
- average of Normal-to-Normal intervals (*avNN*):
  $$avNN=\frac{1}{N}\sum_{n=1}^{N}{NNI}_{n}$$ (7)
- standard deviation of Normal-to-Normal intervals (*sdNN*):
  $$sdNN=\sqrt{\frac{1}{N-1}\sum_{n=1}^{N}({{NNI}_{n}-avNN)}^{2}}$$ (8)
- root Mean Square of Successive Normal-to-Normal interval Differences (*rMSSD*):
  $$rMSSD=\sqrt{\frac{1}{N-1}\sum_{n=1}^{N-1}({{NNI}_{n+1}-{NNI}_{n})}^{2}}$$ (9)
- successive Normal-to-Normal intervals that differ by more than 50 ms (*NN50*):
  NN50=#(NNI>50ms) (10)
- proportion of NN50 divided by the total number of Normal-to-Normal intervals (*pNN50*):
  $$pNN50=\frac{\#(NNI>50ms)}{\#(NNI)}$$ (11)
- proportion of NN20 divided by the total number of Normal-to-Normal intervals (*pNN20*):
  $$pNN20=\frac{\#(NNI>20ms)}{\#(NNI)}$$ (12)

Eq 6, Eq 7 and Eq 8 were also calculated for the RSP signal. As for the EDA signal, the features derived from the skin conductance orienting responses are the following:

- number of Orienting Responses (*nOR*):
  nOR=#OR (13)
- mean magnitude of Orienting Responses (*mmOR*):
  $$mmOR=\frac{1}{nOR}\sum_{n=1}^{nOR}{mOR}_{n}$$ (14)
- mean duration of Orienting Responses (*mdOR*):
  $$mdOR=\frac{1}{nOR}\sum_{n=1}^{nOR}{dOR}_{n}$$ (15)

A sequential feature selection was computed with the extracted features using the MATLAB function *sequentialfs*. This function was applied to all the different classifiers in order to select the most significant feature subset. It starts with an empty feature set and sequentially adds a candidate feature until a given criterion is fulfilled. The criterion for this study was the accuracy of each classifier. Thus, the *sequentialfs* function stopped when there was no further improvement in accuracy.

### Classifier training and validation

The 57 records of our dataset were used for classifier training and validation in MATLAB. The following classification models were compared: decision trees, discriminant analysis (linear and quadratic), k-nearest neighbors, support vector machines, naïve Bayes and ensemble classifiers (Bagged Trees). Decision trees were computed with a maximum number of splits of 100 and the Gini’s diversity index was set as a split criterion. The k-nearest neighbors classifiers were computed with euclidean distance and two different numbers of neighbors (k = 1 and k = 10). Support vector machines were trained with linear and quadratic kernel functions with a box constraint of 1 for both of them. Naïve Bayes were computed with a gaussian kernel. The ensemble classifier type Bagged Trees uses Breiman’s *Random Forest* algorithm [44]. This algorithm is an ensemble of decision tree predictors that randomly splits the training dataset into several subsets. Each subset is trained by different decisions and features, and the result represents the mean of all predictions. Furthermore, according to Breiman, the generalization error of Random Forest converges as the number of trees increases, which makes it more robust against overfitting when compared with individual decision trees. Its options were set to: maximum number of splits = number of observations—1, number of learners = 30, learning rate = 0.1 and subspace dimension = 1.

A 10-fold cross-validation was computed and its outcome provides measures of performance for the different classifiers: Accuracy, True High Rate (THR), True Medium Rate (TMR) and True Low Rate (TLR) were calculated from the confusion matrix. Accuracy is defined by the sum of observations that are correctly classified divided by the total number of observations. Regarding THR, TMR and TLR, they can be defined as the true observations divided by the sum of true observations and false observations. Another typical performance measure is the receiver operating characteristic (ROC) curve. It shows true positive rate (TPR) versus false positive rate (FPR) for each class of the trained classifier.

As additional performance measure, we applied Cohen’s Kappa statistic to the selected trained machine learning algorithms. It compensates for classifications that may be due to chance. The original intent of Cohen [45] was to measure the degree of agreement or disagreement between observers of psychological behavior (known as interrater-reliability). Landis and Koch [46] provided a scheme to interpret Kappa values that vary from -1 to +1: a Kappa value < 0 is indicating no agreement, Kappa values between 0–0.20 are indicating slight, between 0.21–0.40 are indicating fair, between 0.41–0.60 are indicating moderate, between 0.61–0.80 are indicating substantial, and between 0.81–1 are indicating almost perfect agreement. Cohen’s Kappa statistic is a very useful, but under-utilized, measure for comparing the accuracy of classifiers in cases of multi-class and imbalanced class problems [47].

Each of the ten data labeling approaches was applied for the classification of two and of three levels of anxiety, for 19 different combinations of features, and for 18 different variations of classification models. Thus, in total 6,840 different classifiers were trained and evaluated. We selected the favored models by sorting the overall accuracy and taking those with the highest values that, at the same time, have balanced results in the prediction rates of true detection for each class. The purpose of this criteria is to assure high prediction rates for each class disregarding options that could have the highest rates in a particular class while having poor rates for other(s). Moreover, the selection was made by choosing the simplest models, i.e. the ones with the minimum subset of features.

In order to implement a Windows desktop application to test the classifers’ capability for on-line anxiety level detection, four software libraries were created:

- *ECGSignal*: contains ECG signal processing and feature extraction
- *EDASignal*: contains EDA signal processing and feature extraction
- *LowPfilter*: contains low pass filtering for signal processing
- *MLA*: contains the final selection of trained machine learning algorithms

It is worth noting that the RSP signal processing and feature extraction are not included, because most of the RSP features were not useful to improve the accuracy of the models.

## Results

### Subset feature selection

In order to obtain a deeper insight into the relevance of the extracted features tailored to each machine learning algorithm, a sequential feature selection was computed for each algorithm. The recommended feature combinations are shown in the tables of the next section. The subset feature selection was useful for model simplification. The most relevant features are HRNmean, HRstd, EDANmean, EDANFD, EDAnOR, and EDAmmOR. Contrarily, most of the features obtained from the RSP signal were not useful to improve classification results. Even though the normalized mean of the BR appeared to be relevant for a few models, it did not improve the accuracy by much. Thus, in order to simplify not only the models but also the envisioned on-line biofeedback system in a practical manner, the RSP sensor chest strap was disregarded for further steps.

### Evaluation of the machine learning algorithms

Table 3 shows our favored three results on the two-level classification for time windows of 60 seconds and 10 seconds, respectively. Likewise, our favored three results on the three-level classification are displayed in Table 4 for the same time windows. The most frequent classifier in Table 3 is Bagged Trees. HR1 is the most frequent approach while all main approaches are present in this table. The highest accuracy achieved using the HR1 approach is almost 91% for the 60 seconds time window and almost 90% for the 10 seconds time window using the SB approach. The most frequent classifier in Table 4 is Bagged Trees again. SB is the dominant approach while HR2 appears once in this table. Accuracy results are lower than the two-level case but fairly good considering the additional class. The highest accuracy achieved using the SB approach is 73.4% for the 60 seconds time window and 74.4% for the 10 seconds time window using the same approach.

**Table 3:** Favored three results on two-level classification for time-window length of 60 and 10 seconds.

Classifier	Features	Approach	TW [s]	Accuracy [%]	THR [%]	TMR [%]	TLR [%]
Bagged Trees	HRNmean, HRstd, EDANmean, EDANFD, EDAnOR, EDAmmOR	HR1	60	90.9	90.6	-	91.2
Bagged Trees	HRNmean, EDANmean, EDANFD, BRNmean	HR2	60	89.5	91.9	-	85.6
Quadratic SVM	HRNmean, HRNN50, EDANmean	HR1	60	89.1	84.2	-	94.1
Bagged Trees	HRNmean, HRstd, EDANmean, EDANFD, EDAnOR, EDAmmOR	SB	10	89.8	82.4	-	94.7
Bagged Trees	HRNmean, HRstd, EDANmean, EDANFD, EDAnOR, EDAmmOR	HR1	10	89.0	88.4	-	89.6
Bagged Trees	HRNmean, HRstd, EDANmean, EDANFD, EDAnOR, EDAmmOR	EDA2	10	85.3	86.0	-	84.3

Results for time-window length of 60 seconds are shaded light gray.

TW, time window; THR, true high rate; TMR, true medium rate; TLR, true low rate; SVM, support vector machine.

**Table 4:** Favored three results on three-level classification for time-window length of 60 and 10 seconds.

Classifier	Features	Approach	TW [s]	Accuracy [%]	THR [%]	TMR [%]	TLR [%]
Bagged Trees	HRNmean, HRstd, EDANmean, EDANFD, EDAnOR, EDAmmOR	SB	60	73.4	59.3	59.1	92.4
Decision Tree	HRNmean, HRpNN20, EDANmean	SB	60	71.4	46.9	60.9	94.7
Quadratic SVM	HRNmean, EDANmean, EDANFD	SB	60	70.4	35.4	67.8	95.3
Bagged Trees	HRNmean, HRstd, EDANmean, EDANFD, EDAnOR, EDAmmOR	SB	10	74.4	60.0	60.7	93.1
Bagged Trees	HRNmean, HRNN50, EDANmean	SB	10	73.5	60.4	60.6	90.9
Bagged Trees	HRNmean, HRstd, EDANmean, EDANFD, EDAnOR, EDAmmOR	HR2	10	72.3	71.1	71.6	75.2

Results for time-window length of 60 seconds are shaded light gray.

TW, time window; THR, true high rate; TMR, true medium rate; TLR, true low rate; SVM, support vector machine.

Among our favored results, we finally selected among the trained machine learning algorithms by prioritizing the 10 seconds over the 60 seconds time window. We selected five algorithms to have a range of approaches relevant for further test and development (last three rows of Table 3 and row 4 and 6 of Table 4). Table 5 lists the final selection of trained machine learning algorithms for a time window of 10 seconds. All algorithms have the same classifier Bagged Trees and the same combination of two ECG features and four EDA features in common. In addition to the accuracy and the true class rates, we calculated the Cohen’s Kappa as performance measure. All Kappa values show a consistent trend with the accuracy for each algorithm. The algorithms for the two-level classification have Kappa values in the substantial range with two of them, based on the SB and HR1 approach, at the upper limit close to the almost perfect range. The algorithms for the three-level classification, based on the SB and HR2 approach, show Kappa values at the upper limit of the moderate range close to the substantial range.

**Table 5:** Final selection of trained machine learning algorithms for time-window length of 10 seconds.

Classifier	Features	Approach	# Levels	Accuracy [%]	THR [%]	TMR [%]	TLR [%]	Kappa
Bagged Trees	HRNmean, HRstd, EDANmean, EDANFD, EDAnOR, EDAmmOR	SB	2	89.8	82.4	-	94.7	0.78
Bagged Trees	HRNmean, HRstd, EDANmean, EDANFD, EDAnOR, EDAmmOR	HR1	2	89.0	88.4	-	89.6	0.78
Bagged Trees	HRNmean, HRstd, EDANmean, EDANFD, EDAnOR, EDAmmOR	EDA2	2	85.3	86.0	-	84.3	0.69
Bagged Trees	HRNmean, HRstd, EDANmean, EDANFD, EDAnOR, EDAmmOR	SB	3	74.4	60.0	60.7	93.1	0.59
Bagged Trees	HRNmean, HRstd, EDANmean, EDANFD, EDAnOR, EDAmmOR	HR2	3	72.3	71.1	71.6	75.2	0.58

Results for two-level classification are shaded light gray.

THR, true high rate; TMR, true medium rate; TLR, true low rate.

**Fig 6:**
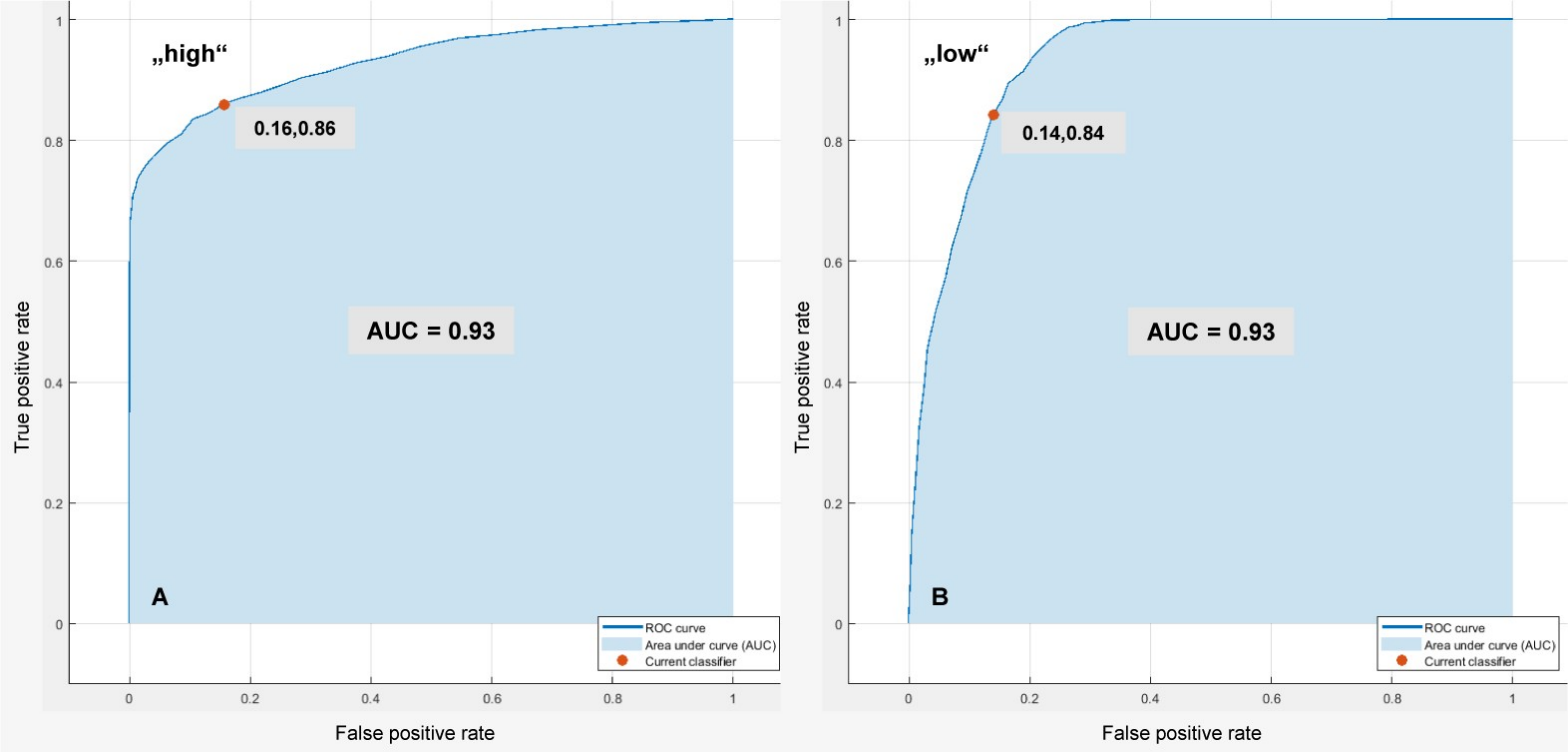
Exemplary plots of ROC curves. Bagged Trees classifier in Table 5 based on the EDA2 approach: (A) Positive class “high”, (B) Positive class “low”.

Fig 6 shows exemplary plots of ROC curves for the Bagged Trees classifier in Table 5 based on the EDA2 approach. The marker on the plot shows the values of the FPR and the TPR for the selected trained classifier. This classifier assigns 86% of the observations correctly and 16% of the observations incorrectly to the positive class ‘high’. Concerning the positive class ‘low’, this classifier assigns 84% of the observations correctly and 14% of the observations incorrectly. The area under curve (AUC) is 0.93 for both.

**Fig 7:**
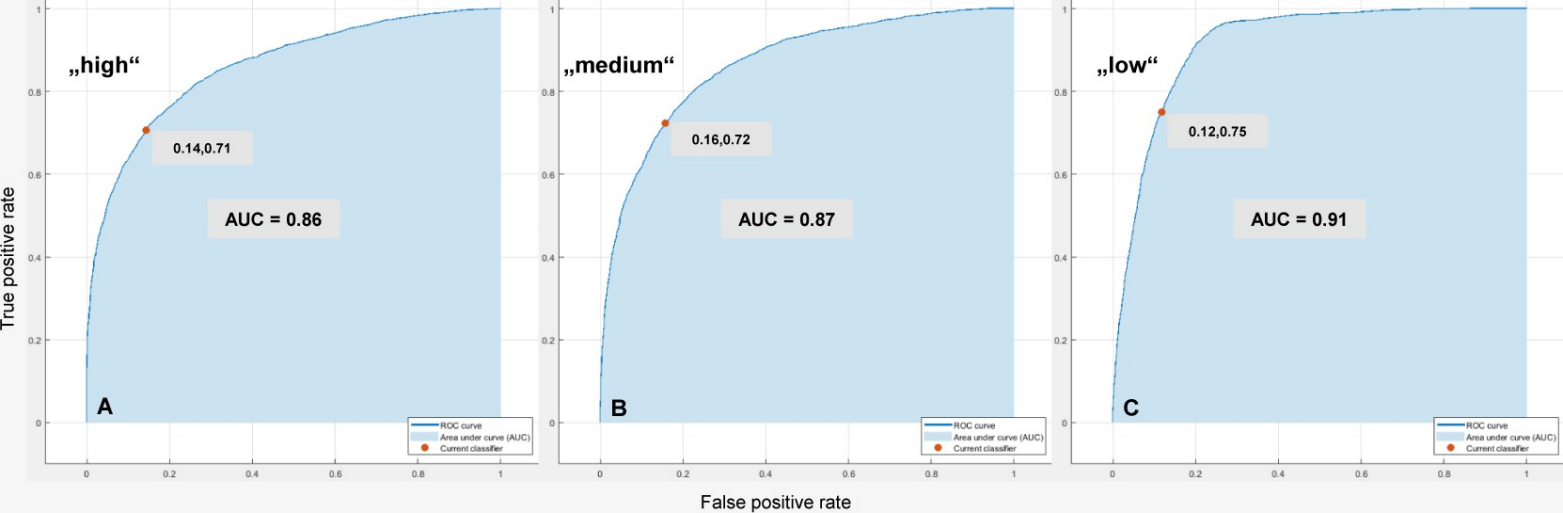
Exemplary plots of ROC curves. Bagged Trees classifier in Table 5 based on the HR2 approach: (A) Positive class “high”, (B) Positive class “medium”, (C) Positive class “low”.

Fig 7 shows exemplary plots of ROC curves for the Bagged Trees classifier in Table 5 based on the HR2 approach. This classifier assigns 71% of the observations correctly and 14% of the observations incorrectly to the positive class ‘high’ (AUC is 0.86). Further, it assigns 72% of the observations correctly and 16% of the observations incorrectly to the positive class ‘Medium’ (AUC is 0.87). Concerning the positive class ‘low’, this classifier assigns 75% of the observations correctly and 12% of the observations incorrectly (AUC is 0.91).

**Fig 8:**
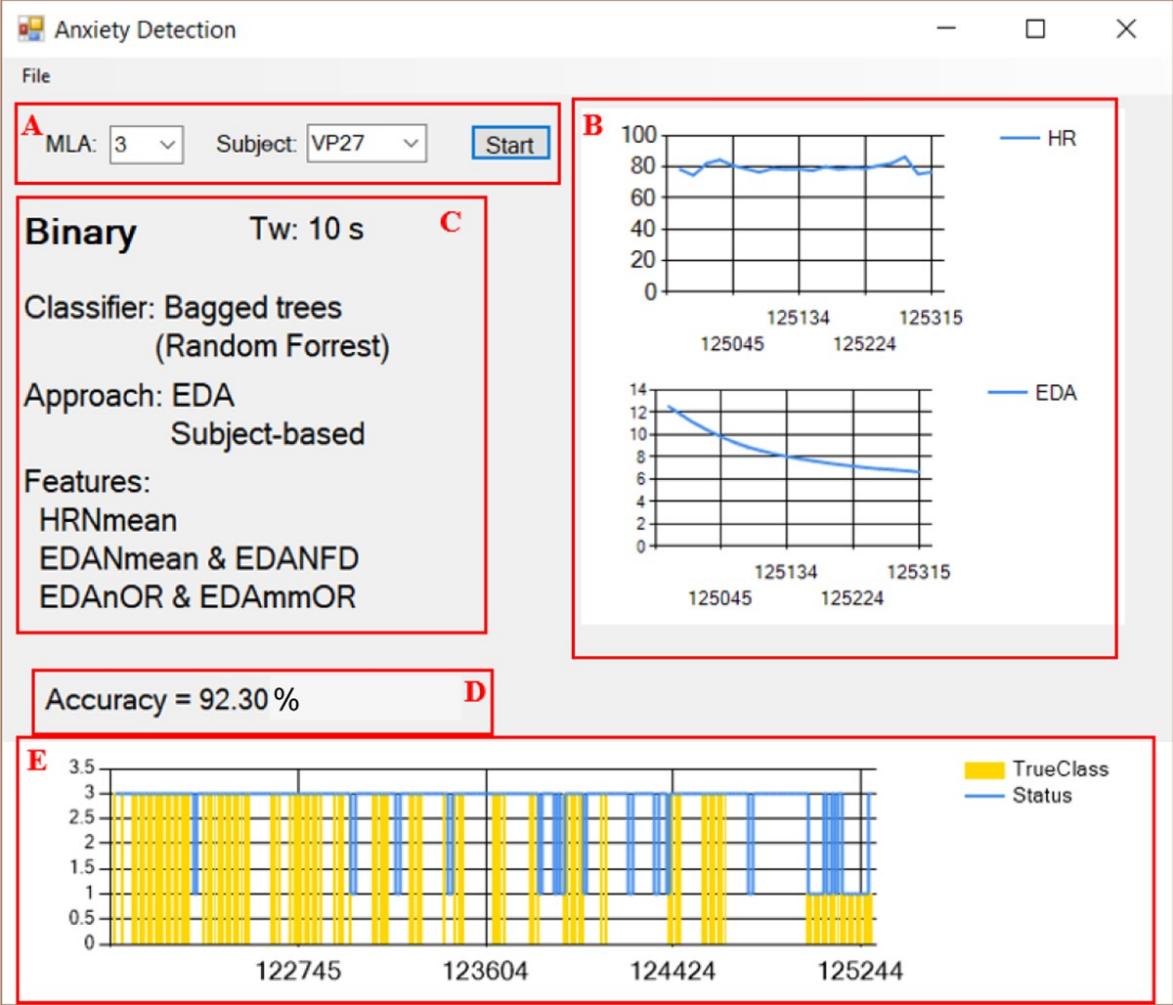
Graphical user interface of the Windows desktop application. (A) Inputs—Machine learning algorithm (MLA) and subject, (B) On-line plots of HR and EDA, (C) Details of the selected algorithm, (D) Calculated accuracy only considering the true classes, (E) On-line prediction status (blue line) and true class according to the labeled data (yellow bars).

First tests with different records of our dataset were carried out in the developed Windows desktop application. In order to test the on-line capability of the trained machine learning algorithms in Table 5, a playback of each record was run, emulating real-time data acquisition. These algorithms were able to classify on-line without any significant delay. Fig 8 shows the graphical user interface of the Windows desktop application.

It is worth noting that the calculated accuracy shown in Fig 8D does not correspond to the accuracy of the classifiers. Also, the true classes depicted in Fig 8E are only displayed when labeled data is played back, as the true classes are known in this case. Thus, the calculated accuracy is only considering the predictions (blue line) corresponding to the true classes (in yellow bars) and the predictions without any true class (no yellow bars) are not taken into account.

## Discussion

Table 6 compares the results obtained in different studies with those of our study. Even though applications are different, classes are quite similar and comparable to our study. For instance, the first four publications in Table 6 are focused on driving-stress detection whereas only Handouzi et al. [32] and our study investigate the treatment of specific phobias. Nevertheless, all cases of application aim for two-level and/or three-level classification.

**Table 6:** Summary of different classifers found in literature in comparison to the results of our study.

Reference	Application	Validation	Features	Classes	Classifier	TW [s]	Accuracy [%]
Healey and Picard [28]	Driving-stress detection	Leave-one-out cross-validation	22, EMG-based EDA-based ECG-based RSP-based Video-based	3 levels of stress	Linear discriminant	300	97.4
Keshan et al. [29]	Driving-stress detection	10-fold cross-validation	1, ECG-based	2 levels of stress	Naïve Bayes	300	100
				3 levels of stress	Neural Networks		70.15
			8, ECG-based	2 levels of stress	Decision trees		97.92
				3 levels of stress	Decision trees		70.15
Chen et al. [30]	Driving-stress detection	Leave-one-out cross-validation	73, EDA-based ECG-based RSP-based + feature selection and reduction	3 levels of stress	SVM	10	89
Barua et al. [31]	Driving-stress detection	72% split, 146 training cases, 58 test cases	IBI-based	2 levels of stress	Case-based reasoning	60	85.63
			Finger temperature				80.45
Handouzi et al. [32]	Social phobia treatment using VRET	71% split, 200 training cases, 80 test cases	6, Blood volume pulse signal	2 levels of anxiety	SVM	20	76
Our study	Arachnophobia treatment using video clips	10-fold cross-validation	6, EDA-based ECG-based	2 levels of anxiety	Bagged Trees	10	89.8
			6, EDA-based ECG-based	3 levels of anxiety			74.4

Results of our study are shaded light gray.

TW, time window.

Other important criteria for a proper comparison are the type of validation, the time window length, and the number of features. The simple split ratio of about 70% from Barua et al. [31] and Handouzi et al. [32] can be considered as least strict method of validation, which is also very prone to overfitting, whereas cross-validation methods are used specifically to avoid overfitting [48]. Although there are known methods that can improve cross-validation, such as the .632+ bootstrap method [49], especially in the case of small datasets [50], we did not apply it in the current stage of this work, because we compared our results with related work in which k-fold cross-validation methods were used. Taking this into account, accuracy rates of our study for two-level and three-level classification are very good as they are within the same range of the accuracy of Barua et al. [31] and Handouzi et al. [32]. Furthermore, we use a similar number of features but a shorter time window in our study. Barua et al. [31] do not specify the exact number of features but mention using several features in time and frequency domain obtained from the finger temperature and the inter-beat-interval (IBI).

The research of Keshan et al. [29] has the same number of folds for the cross-validation. Compared to our findings, their accuracy for the two-level case is higher and the model is simpler (only one feature). Nonetheless, their time window length is 30 times larger (5 minutes vs. 10 seconds) and, for the three-level case, their accuracy is about 4% lower compared to our findings (74.4%). Healey and Picard [28] and Chen et al. [30] applied the strictest validation method. Healey and Picard [28] reported almost a perfect accuracy for their three-level classification, however, the time window is very large (5 minutes) and they included a higher number of features compared to our study. Similarly, the model of Chen et al. [30] using the same time window length is very complex with 73 features in total, however, they obtain a good accuracy of 89%.

Concerning our machine learning implementation, the evaluation of the trained algorithms shows very good accuracy and true class rates as well as substantial Kappa values for the two-level classification. In the case of the three-level classification, the results are lower than the two-level case but fairly good considering the additional class. In contrast to the expectation of a low correlation between physiological and subjective psychological anxiety [27], Table 5 shows that the two algorithms based on the SB approach exhibit very good performance measures that are comparable to those of the HR approaches. However, these algorithms might have learned the *noise* in the training data, which could negatively impact the performance of the algorithm on new data.

Among the two-level classifiers, the most suitable algorithm for further development may be the one based on the HR1 approach. By contrast, the one based on the SB approach was trained with less and uneven data. On one hand, it may not be suitable since it is likely to be overfitted [48]. On the other hand, it should not be omitted for further development due to its substantial Kappa value. The algorithm based on the EDA2 approach is considered because it contains more training data than both other algorithms. Among the three-level classifiers, the most recommendable algorithm may be the one based on the HR2 approach, since the other one is based on the SB approach. Thus, the two algorithms presented in Table 5 based on the HR approaches may be proposed as final recommendation for two-level and three-level anxiety classification.

For future research, it is recommendable to analyze the feasibility of a self-trained algorithm. Firstly, the system could start with the same pre-trained algorithm and, as the therapeutic game sessions go on, it could gather new data and retrain itself. Such an outcome is expected to be more tailored to individual features and thus, more accurate. On the other hand, the drawback of this idea is that it requires storage for both, the pre-trained data and the new data. Moreover, the new data could also be unfavorable if self-help or minimal-contact therapy is not performed properly by the individual. Hence, some trials will be necessary to examine the balance between the improvement in accuracy and computational implications.

Complementary to previous recommendations, having more data would allow exploring the area of deep learning, which is an emerging field in machine learning [51], and investigating the possibility of implementing more classes, i.e. more levels of anxiety. This would provide the VRET/ARET system with more degrees of freedom to modulate the intensity of the sessions.

## Conclusion

The current study describes training and validation of supervised machine learning algorithms for two-level and three-level classification of anxiety. The results show that Bagged Trees is the most suitable classifier among the classification models studied. We discovered remarkable performance measures for both classification cases that are comparable to similar research. The trained machine learning algorithms will have practical impact on the feasibility study of a VRET/ARET system for the treatment of arachnophobia. In this study, the performance of these algorithms on new data will be investigated. This might further inform a decision which data labeling approach should be favored for such and similar applications. Moreover, the main contributions of the current study can be summarized as follows:

- Simple and efficient algorithms with a minimum subset of six features.
- On-line classification with an adequate short time window of 10 seconds.
- Overall high accuracy, high and balanced true class rates as well as good Kappa values.

Technology-based self-help and minimal-contact therapies have been proposed as effective and low-cost interventions for anxiety and mood disorders in recent years. In fact, available state-of-the-art, commercial technologies such as VR/AR glasses and wearable sensors permit the continuous acquisition of real-time data and the use of such data for individualized treatments.

The underlying research question is whether biofeedback on anxiety levels could be used as a beneficial therapeutic add-on for exposure treatment with the aim to increase and to stabilize its effects. This question should be addressed in future studies.
